# Coronavirus diagnosis using cough sounds: Artificial intelligence approaches

**DOI:** 10.3389/frai.2023.1100112

**Published:** 2023-02-15

**Authors:** Kazem Askari Nasab, Jamal Mirzaei, Alireza Zali, Sarfenaz Gholizadeh, Meisam Akhlaghdoust

**Affiliations:** ^1^Materials Science and Engineering Department, Sharif University of Technology, Tehran, Iran; ^2^Infectious Disease Research Center, Department of Infectious Diseases, Aja University of Medical Sciences, Tehran, Iran; ^3^Infectious Disease Research Center, Shahid Beheshti University of Medical Sciences, Tehran, Iran; ^4^Functional Neurosurgery Research Center, Shohada Tajrish Comprehensive Neurosurgical Center of Excellence, Shahid Beheshti University of Medical Sciences, Tehran, Iran; ^5^USERN Office, Functional Neurosurgery Research Center, Shahid Beheshti University of Medical Sciences, Tehran, Iran; ^6^Civil Engineering Department, Tehran University of Technology, Tehran, Iran

**Keywords:** coronavirus, cough, artificial intelligence, machine learning, respiratory sounds, deep learning

## Abstract

**Introduction:**

The Coronavirus disease 2019 (COVID-19) pandemic has caused irreparable damage to the world. In order to prevent the spread of pathogenicity, it is necessary to identify infected people for quarantine and treatment. The use of artificial intelligence and data mining approaches can lead to prevention and reduction of treatment costs. The purpose of this study is to create data mining models in order to diagnose people with the disease of COVID-19 through the sound of coughing.

**Method:**

In this research, Supervised Learning classification algorithms have been used, which include Support Vector Machine (SVM), random forest, and Artificial Neural Networks, that based on the standard “Fully Connected” neural network, Convolutional Neural Networks (CNN) and Long Short-Term Memory (LSTM) recurrent neural networks have been established. The data used in this research was from the online site sorfeh.com/sendcough/en, which has data collected during the spread of COVID-19.

**Result:**

With the data we have collected (about 40,000 people) in different networks, we have reached acceptable accuracies.

**Conclusion:**

These findings show the reliability of this method for using and developing a tool as a screening and early diagnosis of people with COVID-19. This method can also be used with simple artificial intelligence networks so that acceptable results can be expected. Based on the findings, the average accuracy was 83% and the best model was 95%.

## 1. Introduction

The COVID-19 pandemic is caused by the SARS-CoV-2 virus (Laguarta et al., 2020; Jamshidi et al., 2021; Moazzami et al., 2021; Haritaoglu et al., 2022; Samieefar et al., 2022). This disease was detected for the first time in December 2019 in Wuhan, Hubei Province, China (Laguarta et al., 2020; Jamshidi et al., 2021; Liu et al., 2021; Moazzami et al., 2021; Samieefar et al., 2022). On March 11, the World Health Organization (WHO) declared the COVID-19 pandemic (Laguarta et al., 2020; Liu et al., 2021; Chadaga et al., 2022). COVID-19 disease spreads to other people through tiny respiratory droplets (Wang and Wong, 2020; Liu et al., 2021; Chadaga et al., 2022). Studies show that one of the most common symptoms in COVID-19 is dry cough with a prevalence of about 68% (Bai et al., 2020; Wang and Wong, 2020; Mohammed et al., 2021).

During the peak of the spread of COVID-19, medical diagnostic laboratories and health centers face many problems with current common methods such as clinical examinations, computerized tomography (CT) scans, real-time polymerase chain reaction (PCR) and serology techniques (Wan et al., 2016; Ai et al., 2020; Guan et al., 2020).

Despite the fact that real-time PCR is an accurate method for detecting COVID-19, it has some drawbacks. Its major drawbacks include the high cost of testing and the time-consuming testing process, which makes all members of society unable to access it (Cabitza et al., 2020; Xiao et al., 2020). Due to the similar effects of influenza and COVID-19 on the lung, diagnosis based on CT scan images is difficult, and the virus may not infect the lung or maybe due to the imaging done before the third day of infection with COVID-19 the lung infection cannot be detected in the CT scan images (Bleier and Welch, 2020; Khorramdelazad et al., 2021). It should be noted that CT scan is prohibited in infants and pregnant women (Li and Xia, 2020), therefore CT scan imaging during the period of the spread of COVID-19 cannot be an accurate diagnostic method and its use has limitations. Serological tests such as C-Reactive Protein (CRP) indicate any viral infection in the body and are not specific for the COVID-19 virus (Wang, 2020). Also, clinical symptoms are different in people and due to the similarity of the symptoms of COVID-19 with other diseases such as cold and flu, the doctor may make a mistake in diagnosis and this mistake can have adverse consequences for the patient (Struyf et al., 2020).

Other problems include limited medical and health staff, the lack of raw materials and diagnostic devices and in different cities and countries, and the burnout of personnel due to the high number of samples and the time-consuming nature of the testing process (Pooladi et al., 2020). All these problems reduce accuracy and increase errors in diagnosis, which can have very adverse effects on patients and the country's health system, Therefore, one of the ways to solve the problem is to use artificial intelligence, artificial intelligence can significantly reduce the error caused by the accuracy and duration of the detection process (Naudé W., 2020; Soltani et al., 2022).

By using the anatomy of the respiratory system, it is possible to measure the amount of changed respiratory infections based on the cough sound (Bai et al., 2020; Alqudaihi et al., 2021; Mohammed et al., 2021). In the past years, studies have been conducted to identify whooping cough (pertussis), chronic obstructive pulmonary disease (COPD), tuberculosis and asthma using an algorithm of audio signals by analyzing cough sounds (Bai et al., 2020; Alqudaihi et al., 2021; Mohammed et al., 2021; Sadhana et al., 2021; Santosh et al., 2022). Nowadays, some universities in the world, including MIT University in the United States, Cambridge University in England, EPFL University in Switzerland, and Carnegie Mellon University in United States are studying the diagnosis of COVID-19 through cough with the help of artificial intelligence methods (Bai et al., 2020; Wang and Wong, 2020; Alqudaihi et al., 2021; Mohammed et al., 2021). It has been reported that simple machine learning tools, such as binary classifiers, can distinguish COVID-19 breath sounds from healthy counterparts with an area under the ROC curve (AUC) >0.80 (Brown et al., 2020). There appeared to be unique patterns in the COVID-19 coughs that allowed the pre-trained Resnet18 classifier to identify the COVID-19 coughs with an AUC of 0.72. In this case, cough samples were collected by telephone from 3,621 people with confirmed COVID-19 (Bagad et al., 2020).

A high AUC of more than 0.98 was also reported when distinguishing COVID-19-positive from COVID-19-negative coughs in a clinically validated dataset of 2,339 COVID-19-positive and 6,041 COVID-19-negative cases using classifiers based on DNN was obtained (Andreu-Perez et al., 2021).

Comparison of chest CT scans of people with pneumonia not related to COVID-19 and pneumonia related to COVID-19 shows that the possibility of peripheral distribution, ground-glass opacities and thickening of vessels is more in pneumonia related to COVID-19. These findings shows that cough sounds with pneumonia caused by COVID-19 probably have some specific characteristics that result from the fundamental pathomorphological changes (Wang and Wong, 2020). Coughs of respiratory syndromes such as COVID-19 have hidden and specific characteristics, even if they are not spontaneous (Bai et al., 2020). Therefore, cough can be used as a pre-screening method. However, Because of that cough is a symptom of more than 30 different diseases, it is difficult to diagnose COVID-19 through the analysis of cough sounds using artificial intelligence (Bai et al., 2020; Mohammed et al., 2021).

In our research, we went to people's cough because there were the following reasons for this: The sound of people's cough is less diverse than the accent and dialect that is present in speaking. Two factors, the fact that the sound of coughing is more involuntary than talking and that coughing is part of the body's natural mechanism, make the role of accents and dialects, culture and geographical location in coughing less. Easier to collect cough data than other types of data. Easier to train people to use this method. Our work differs from these works in data collection, as we use an entirely crowdsourced dataset, the following are significant and important, Even though some datasets are publicly available, the datasets are naturally limited in COVID-positive samples compared to the negative samples. The collected sounds were collected through the website and online, and there are no direct recorded sounds by offline recorders. The recorded sounds are not from a specific type of microphone, but by different types of smartphones, tablets and laptops, which have different brands and have different browsers. This can have a positive effect on not being limited to one type of microphone to avoid bias and single device. we must further overcome the challenges of data coming from different phones and microphones, possibly in very different environments.

In all the above cases, due to the fact that we have been able to obtain a high amount of data, we were able to eliminate the effects of the lack of data for training our models and achieve acceptable results.

The purpose of this study is to create data mining models in order to diagnose people with the disease of COVID-19 through the sound of coughing. This study was designed for the first time in Iran, and its most important advantage is the rapid and non-invasive diagnosis of COVID-19.

## 2. Methods

Since the deficiency of data can cause overfitting, we increased the data (data augmentation) to be learned by artificial intelligence with a standard method. According to the data augmentation standard, the data should be changed in such a way that the quality and nature of the data do not change, because it can cause a fundamental change in the data. In the data used in model training, the speed of the audio signal was changed with different rates (0.8–1.10 times) and the pitch of the audio signal was changed with different steps (−2.5 to 2.5 times). There was no change in the test data.

### 2.1. Data collection

From January 2021, data related to cough of people with COVID-19 was recorded and collected using the online site “sorfeh.com/sendcough/en.” In order to increase the number of data, the contact information of patients with COVID-19 was received through laboratories, hospitals and infectious specialists. During this process, the data was collected after the patient's consent and having the required conditions. The conditions for the patient to enter the study include a positive PCR test, or CT Scan diagnosis of lung involvement by CT scan, or a definite diagnosis of corona virus by a physician based on clinical examinations, and no more than 8 days have passed since the definite diagnosis of COVID-19. People admitted to the hospital were excluded from the study because usually more than 8 days have passed since the duration of the illness of the hospitalized people and their coughs could be pulmonary complications after COVID-19 and not related to COVID-19.

Inclusion criteria for healthy people into the study: (1) lack of symptoms of the COVID-19, (2) if the symptoms are present, the physician's diagnosis should not be COVID-19, and (3) None of the close people should have COVID-19 (Alqudaihi et al., 2021; Sadhana et al., 2021; Santosh et al., 2022). During data collection and recording of coughing, healthy people and patients were asked to cough in a safe environment without the presence of other people.

### 2.2. Data collection app

**Figure 1:**
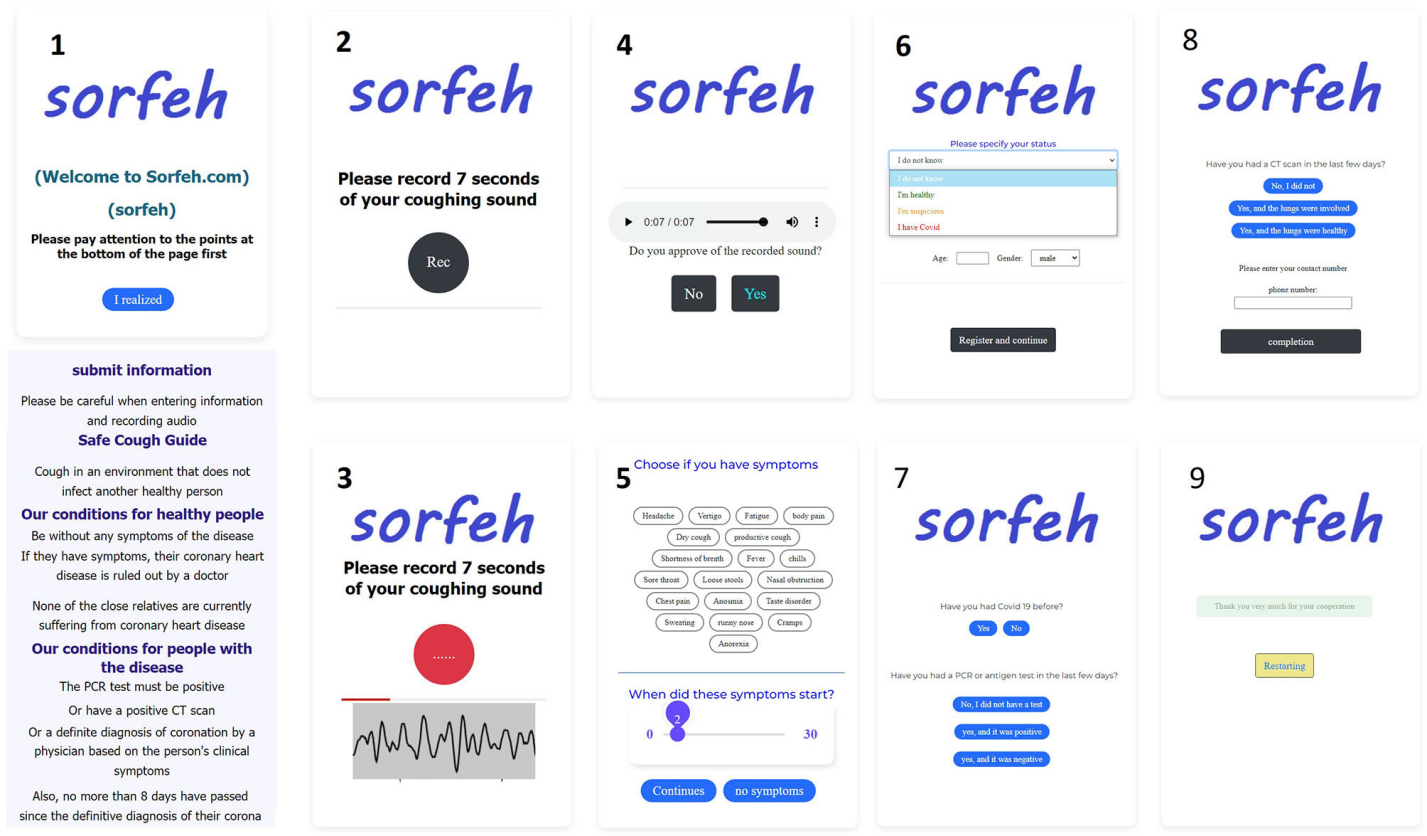
How to collect data and its steps on the website.

The link of the online site was provided to the patients. Patients first chose their symptoms on the online site and recorded their cough for 7 s in a quiet environment and without the presence of people. The presence of sounds other than coughing can cause misdiagnosis (Figure 1). Then the recorded sound will be played and if the quality is confirmed, it will go to the next stage. In the next step, the user must select the symptoms he has and select the time of occurrence. Then he will be asked additional questions, such as the current status, age, gender, previous history of infection, PCR test, CT scan of the lung, whether there is a conflict or not. The recorded sounds are not from a specific type of microphone and have been recorded by different types of smartphones, tablets and laptops. This case can have a positive effect on the study and avoid bias. All the data collected are from Tehran city, which are from different minorities and ethnicities with various accents. All audio files used in our study are in uncompressed Pulse-code modulation (PCM) 16-bit format with a sampling rate of 48 kHz and a fixed 7-s length (Pahar et al., 2022).

### 2.3. Crowdsourced dataset

The total number of collected data is almost 40,353. Almost 17,690 data were manually removed due to incomplete information, low sound quality, silent audio content, presence of surrounding noise when recording cough, absence of cough sound in audio content. Among the remaining 22,663 cough sounds, there were 14,521 negative coughs and 8,142 positive coughs. On the website, in addition to recording the sound of coughing, patients also recorded information such as clinical symptoms, days of onset of symptoms, the status of PCR tests and CT scans, the presence of previous infections, age, gender, and the person's disease status. The information about the symptoms of the patients is shown in Table 1. According to Table 1, the most common symptom among people with COVID-19 is fever (51.75%). Dry cough and productive cough are among the most common symptoms of the patients participating in the study with prevalence of 25.01 and 21.22%. Also, 29.2% of patients had no clinical symptoms. The symptoms of healthy people were negligible, so it was ignored.

**Table 1:** Distribution of symptoms of COVID-19 patients.

**Symptom**	**Percentage**	**Number**
Fever	51.75	4,214
Dry cough	25.01	2,037
Productive cough	21.22	1,728
Fatigue	19.83	1,615
Body pain	17.31	1,410
Sore throat	15.99	1,302
Loose stools	12.29	1,001
Runny nose	11.05	900
Vertigo	3.16	258
Shivering	9.83	801
Sweating	9.03	736
Dyspnea	8.64	704
Abdominal pain	4.27	348
Nasal obstruction	3.13	255
Taste disorder	3.09	252
Chest pain	2.03	166
Olfactory disorder	2.01	164
Anorexia	1.98	162

The age range and gender of patients and healthy people are also mentioned in Table 2. The age range of the participants was 5–90 years, most of the participants were between 15 and 30 years old and most of them were women. All participants were residents of Tehran city, which are from different minorities and ethnicities with various accents.

**Table 2:** Age and gender distribution of participants.

**Characteristics**		**Number of patients**	**Number of healthy people**
Number of participants		8,142	14,521
Gender	Male	2,301	4,649
	Female	5,841	9,872
Age range	5–15	754	1,240
	15–30	3,836	4,151
	30–45	2,241	1,985
	45–60	946	725
	60–75	326	578
	75–90	39	47

### 2.4. Dataset used for this analysis

The analysis will only be done on the classification and diagnosis of the infected and healthy person based on the sound of the cough, so no classification was done on each of the files of each category of coughs. We used all the positive samples of COVID-19 in our data set and randomly used the same number of negative samples of COVID-19 for a balanced distribution of samples (8,142 person in each group). The patients in the case group were selected with the conditions of (1) a positive PCR test, or (2) diagnosis of lung involvement by CT scan or (3) definite diagnosis of corona virus by a physician based on clinical examinations, and (4) no more than 8 days have passed since the definite diagnosis of COVID-19 and healthy people were selected in the control group with the conditions of (1) lack of symptoms of the COVID-19, (2) if the symptoms are present, the physician's diagnosis should not be COVID-19, and (3) none of the close people should have COVID-19.

### 2.5. Feature extraction

We used Handcrafted Features to extract audio features. The frequency of the recorded raw audio was 48 kHz and was stored without compression. We used the librosa library to extract features. Extracting more features does not always lead to beneficial results, so first we extracted the available features according to their previous applications in medical diagnosis. If no result is obtained, more features are extracted.

**Figure 2:**
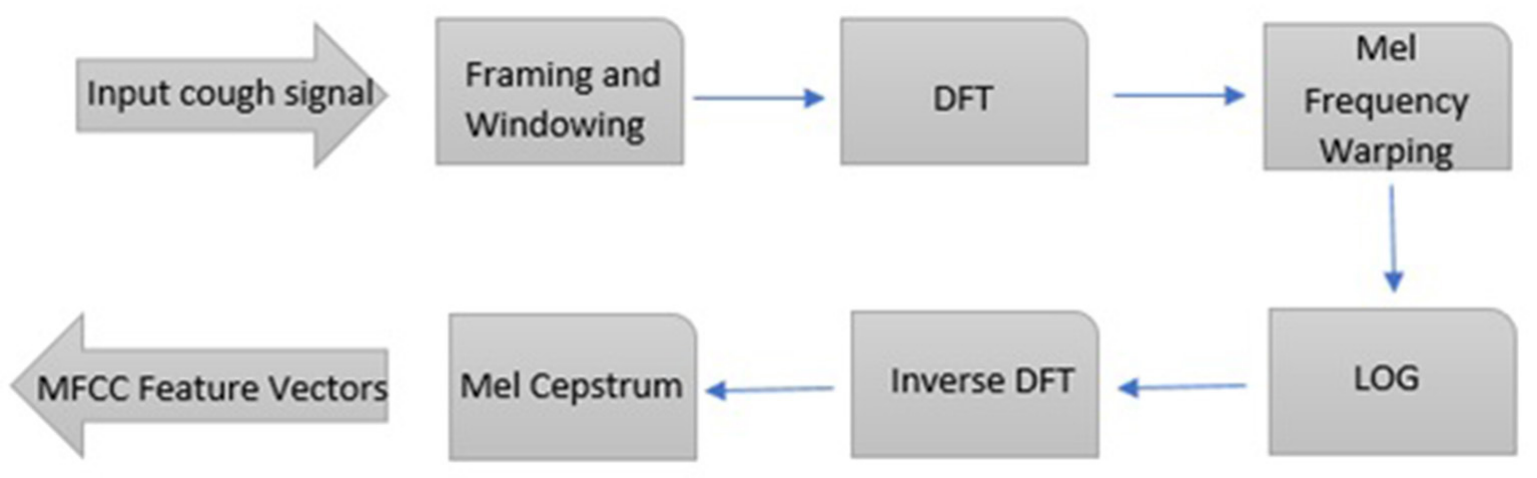
Steps of capstral conversions of the signal spectrum. Steps to obtain mel-scale capstral coefficients from an audio file.

The most common and useful features for speech and audio recognition are Mel-Frequency Cepstral Coefficients (MFCC). This feature can create higher resolutions at lower frequencies. The main idea in extracting MFCC coefficients is the property of the human ear in receiving and understanding speech, and this issue has made these coefficients a powerful tool in all areas of audio processing and recognition. The number of coefficients used in voice recognition usually varies between 9 and 13. The coefficient of 0 indicates energy, which is referred to as the characteristic of Shimmer. In the first step of extracting these coefficients, the Fourier is converted into a signal. Then the obtained spectrum exponent is expressed in mel scale and logarithm is taken from the exponent at each mel frequency. In the last step, the logarithmic spectrum of Mel is returned to the time domain. The result of these transformations is the Capstral representation of the signal spectrum, which shows the spectral characteristics of a frame of the audio signal (Figure 2).

We apply the Discrete Fourier Transform (DFT) on each cough audio (Picone, 1993).


(1) $$\begin{array}{l} Y\left(k\right)=\overset{N-1}{\underset{t=0}{\sum }}y_{i}\left[t\right]w\left(t\right)\exp\left(-2\pi ikt/N\right),k=0,1,...,N-1 \end{array}$$

Where *N* denotes the number of samples in frame, *y*_*i*_*[t]* is the discrete time domain cough signal, *w(t)* is the window function in time domain and *Y(k)* is the kth harmonic corresponding to the frequency.

*f* (k) = k*F*s/*N* where Fs is the sampling frequency. MFCCs use Mel filter bank or triangular bandpass filter on each cough audio DFT output, equally spaced on the Mel-scale. At last, we apply the Discrete Cosine Transform (DCT) on the output of the log filter bank in order to get the MFCCs:


(2) $$\begin{array}{l} c\left(i\right)=\sqrt{\frac{2}{M}}\overset{M}{\underset{m=1}{\sum }}\log\left(E\left(m\right)\right)\cos\left(\frac{\pi i}{M}\left(m-0.5\right)\right) \end{array}$$

Where *i* = 1, 2, …, l; l denotes the cepstrum order, *E(m)* and M are the filter bank energies and total number of mel-filters, respectively. An audio signal is constantly changing, so for ease of understanding, it is assumed that on short time scales the audio signal does not change statistically. For this reason, we frame the signal in 10–100 ms frames. If the frame is too short, we won't have enough samples to get a reliable spectral estimate, and if it's too long, the signal will change too much over the entire frame. Mel's scale was used to more accurately determine which frequency is present in the frame. The Mel scale relates the perceived frequency or pitch of a pure sound to its actual measured frequency. Using this scale makes the features more consistent with what humans hear.

In extracting features, we first set pre-emphasis equal to 0.97 and normalize audio for each audio file, and in extracting features, in addition to MFCC, we considered delta and delta2 MFCC. Where delta MFCC is the temporal differential (delta) of the MFCC and delta2 MFCC is the differential of the delta of the MFCC (acceleration coefficients). The number of coefficients considered 13 and 23 ms step was used to generate MFCC coefficients, and the number of 302 frames was obtained, and average numbers were used in each frame.

Also, during to 7 s considered extracting chroma stft, rmse, spectral centroid, spectral bandwidth, rolloff, zero crossing rate, tonnetz and melspectro cases. Finally, 914 features were obtained (3 ^*^ 302 + 8 = 914).

Where (chroma stft) is chromagram from a waveform or power spectrogram and (rmse) is the root-mean-square of the magnitude of a short-time Fourier transform which provides the power of the signal. (Spectral centroid) is the mean (centroid) extracted per frame of the magnitude spectrogram. (Spectral bandwidth) is the band width of light at one-half the peak maximum. (Rolloff) is the center frequency for a spectrogram bin so that at least 85% of the energy of the spectrum in this frame is contained in this bin and the bins below. (Zero crossing rate) is the rate of sign-changes of the signal. (Tonnetz) is tonal centroid features and (melspectro) is mel-scaled spectrogram.

### 2.6. Data exploration

**Figure 3:**
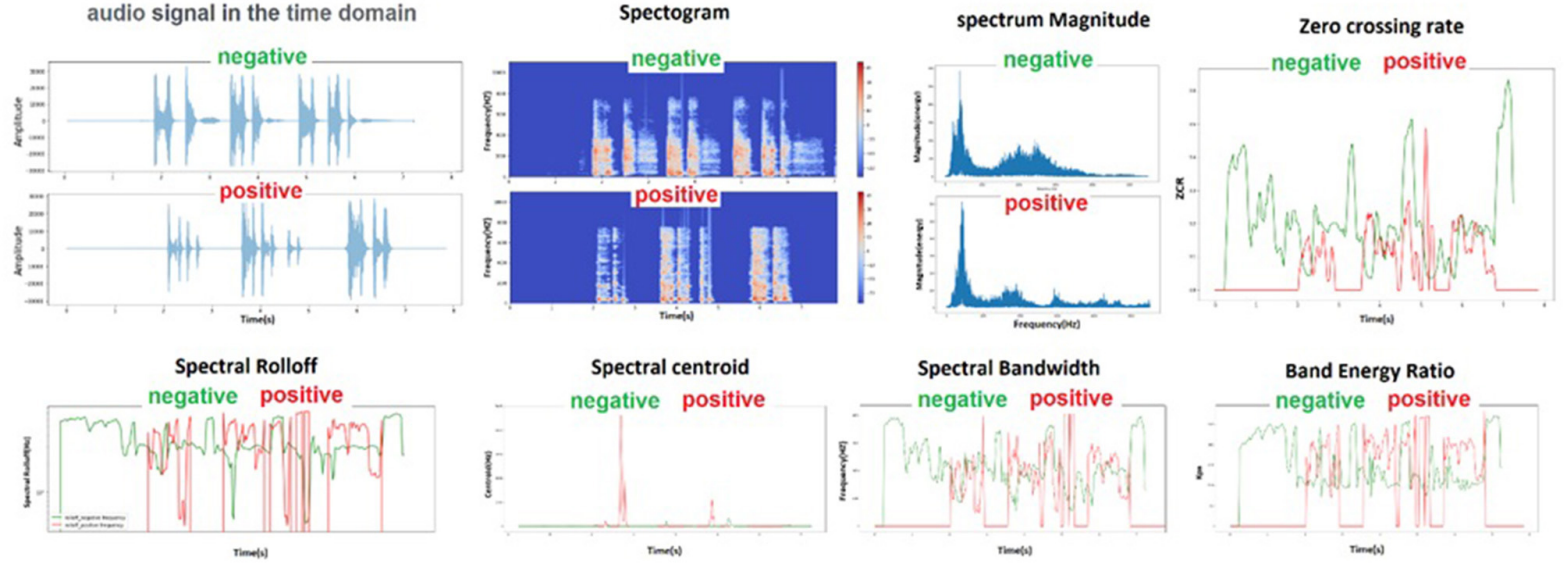
Schematic of audio characteristics for two audio file samples of a healthy and an infected person that were randomly selected.

From each control and case group, one sample was selected that were similar in terms of the number of coughs and phonetics, and their extracted characteristics are shown in Figure 3.

**Figure 4:**
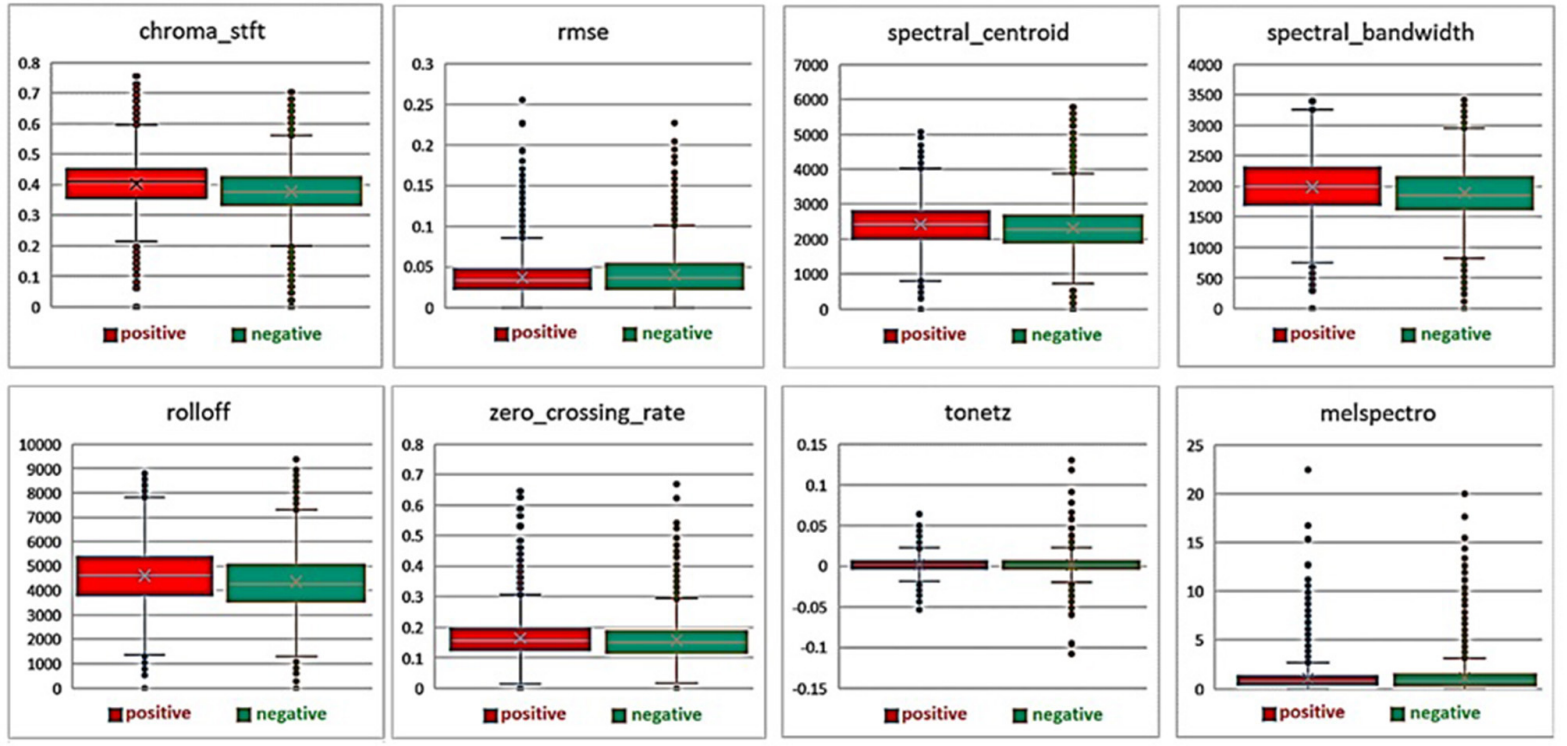
Average, minimum and maximum values of features extracted from healthy and diseased groups.

Twenty-three milliseconds step was used to generate MFCC coefficients, and the number of 302 frames was obtained, and average numbers were used in each frame. After generating the MFCC coefficients, they can be plotted on a spectrogram to visualize the sound. Heat maps can easily distinguish between two groups. In the healthy and sick group, the minimum, maximum and average values of the features that had a significant difference after extraction are shown in Figure 4.

## 3. Evaluation

### 3.1. Experimental setup

In this study, after the data was collected and the initial pre-processing was done, the dataset of the samples was prepared in two train and test groups. Then, data augmentation was done using standard methods, and finally common and useful features were extracted from them.

We trained our models in two modes. The first case is the use of primary data without augmentation the data between two categories of positive and negative COVID-19 and the second case is the use of augmentation data that has been increased for both positive and negative COVID-19 categories.

We have trained and evaluated five machine learning classifiers in total. Supervised Learning classification algorithms have been used, which include Support Vector Machine (SVM), random forest, SVM classifiers have performed well in classifying cough events (Sharan et al., 2017). Also the artificial neural networks that were based on the standard “Fully Connected” neural network, Convolutional Neural Networks (CNN) and Long Short-Term Memory (LSTM) recurrent neural networks have been established. CNN is a popular deep neural network architecture primarily used in image classification. It has also performed well in the classification of breathing and speech of COVID-19 (Pahar and Niesler, 2021). An LSTM model is a type of recurrent neural network whose architecture allows it to remember previously-seen inputs when making its classification decision. It has been successfully used in automatic cough detection (Miranda et al., 2019).

We tested different techniques for creating classification models, first to examine how effective these methods were for classification ability, and finally to examine the success rate in each mode to see which type of classification more efficiency can be achieved.

The independent term in the kernel functions is chosen as a hyperparameter during the optimization of the SVM classifier. The networks was optimized using the *Cross-Entropy Loss* and *Adam optimizer* with default parameters.

We selected several standard evaluation measures such as F1-Score, Sensitivity/Recall, Specificity, Precision and Accuracy for each model to evaluate the best models and their results. Several iterations were performed and the results reported. Table 3 shows the results of each model with the following observations: It was observed that most of the models have accuracy, sensitivity and specificity higher than 70% and this shows that cough provides the necessary data about the respiratory system and the pathogens involved. The signal processing features enable the model to capture latent cough sounds and detect COVID-19 with sufficient sensitivity and specificity.

**Table 3:** Results of each model before and after augmentation data.

**Model base**	**F1-Score (%)**		**Sensitivity (%)**		**Specificity (%)**		**Precision (%)**		**Accuracy (%)**	
	**Before**	**After**	**Before**	**After**	**Before**	**After**	**Before**	**After**	**Before**	**After**
SVM	81.98	83.56	82.96	85.57	80.63	80.83	81.01	81.64	81.8	83.2
Random-forest	62.27	74.19	64.83	75.75	59.22	71.65	59.91	72.69	61.94	73.7
Fully connected	81.23	86.16	81.56	87.97	80.8	83.83	80.91	84.42	81.2	85.9
CNN	76.76	82.17	77.15	83.16	76.24	80.83	76.38	81.21	76.7	82
LSTM	89.06	95.01	90.65	95.50	87	94.71	87.52	94.53	88.83	95.1

Although all the models were able to achieve the minimum required evaluations, but not all of them are necessarily acceptable, and in the existing conditions, each of them shows different functions, and the best performance in the final use of the model and method should be considered. It can also be seen that by using the data augmentation method, most of the parameters related to the evaluation of the models have been improved.

## 4. Discussion and conclusions

The results of the study by Zhao and et al. and Li and et al. show that the three symptoms of fever, dry cough and fatigue are the most common symptoms of COVID-19 patients (Li et al., 2020; Zhao et al., 2020). Based on the results of this study, fever (51.75%), dry cough (25.01%) and fatigue (19.83%) were among the 3 most common symptoms of patients (Table 1). The report of the World Health Organization also confirms the results of the studies (World Health Organization., 2020).

After collecting about 40,000 data and initial processing, the data were divided into test and training groups. Then, in order to strengthen the data, data augmentation was done in a standard method. in this study, Supervised Learning classification algorithms have been used, which include Support Vector Machine (SVM), random forest, and also the artificial neural networks that were based on the standard “Fully Connected” neural network, Convolutional Neural Networks (CNN) and Long Short-Term Memory (LSTM) recurrent neural networks have been established, and we selected several standard evaluation measures such as F1-Score, Sensitivity, Specificity, Precision and Accuracy for each model to evaluate the best models and their results. Accuracy, the percentage of correct identification and diagnosis of the system, and sensitivity, the percentage of the diagnosis of infected people shows that the higher the percentage of sensitivity, the more number of infected people will be identified (Sharma et al., 2021). In this study, after increasing the data, the accuracy of all models increased. before increasing the data, the average accuracy of the models was 78%, and after increasing the data, the average accuracy reached 83% and the best model was 95% (Table 3).

In the present study, a favorable sensitivity was considered for the participants, and with the average accuracy obtained, we hope to be able to identify more patients in the society with this screening method in order to reduce the rate of COVID-19 infection and prevent its increase. Companies and organizations that are more sensitive to the disease of COVID-19, can consider a higher sensitivity to identify more patients using this screening method. If the sensitivity increases in the fixed accuracy level, healthy people may also be mistakenly included in the group of patients, but because the purpose of this method is to reduce the rate of infection, this method will be of higher quality. We propose a triaging tool that could be used by both individuals and health care officials.

The possibility of unwanted entry of some patients into the control group and vice versa is one of the most important limitations of this study (Alsharif et al., 2020; Ghose et al., 2020; Wong et al., 2020; van Ginneken, 2021). A person may be in a stage of COVID-19 disease that has no clinical symptoms or is not infected with COVID-19 but the lungs are affected due to other respiratory complications or smoking. It is also possible that the person has coughed several times while recording the cough sound, which causes inflammation of the person's larynx (Ghose et al., 2020). However, increasing the number of data can lead to better discrimination and higher accuracy.

The implementation of this screening and diagnostic method at the community level can lead to useful results. The importance and benefits of conducting this study include reducing the workload of medical and health staff, especially during the peak of the outbreak of COVID-19, identifying more patients and reducing diagnostic and treatment costs, especially in less developed countries. Diagnosis and screening in this method is very simple and people will not have any worries about doing it, so more patients can be identified and prevent the spread of the disease. Also, according to the diagnostic method of this study, the workload of health and treatment staff is reduced and the staff can devote their time and energy to treating patients, which can also have a positive effect on increasing the number of recoveries of COVID-19 patients.

In order to use this method more effectively in medicine, it seems necessary to conduct more studies to distinguish between the cough sounds of COVID-19 patients and other people with lung problems and complications.

## Data availability statement

The raw data supporting the conclusions of this article will be made available by the authors, without undue reservation.

## Author contributions

Conceptualization of this study by SG, and took the lead in writing the manuscript in consultation with MA. JM and AZ assisted with the calculations, technical details, and drafting the manuscript. SG, JM, and AZ helped to write the manuscript. All authors contributed to the article and approved the submitted version.
